# ID 172 - Associação dos Implantes Mamários de Superfícies Texturizada e Lisa ao Linfoma Anaplásico de Células Grandes (alcl): uma revisão sistemática

**DOI:** 10.5327/2237-9622.2025.v34s1.172

**Published:** 2025-11-25

**Authors:** Cristiane Rocha de Oliveira, Grasiela Martins da Silva, Quenia Cristina Dias Morais, Verônica Clemente

**Keywords:** implantes mamários, linfoma não-Hodgkin, BIA-ALCL

## Abstract

**Introdução:**

Em 2011, ocorreu suspeita do FDA da associação de implantes mamários com uma forma rara de linfoma não-Hodgkin, o linfoma anaplásico de células grandes ALCL. A OMS reconheceu e definiu em 2016 a condição clínica como linfoma associado a implantes mamários (BIA-ALCL). Em 2018, a agência francesa ANSM suspendeu a comercialização dos implantes texturizados da empresa Allergan com a perda do Certificado de conformidade por possível associação com BIA-ALCL. A Agência Nacional de Vigilância Sanitária (Anvisa) publicou a Resolução n.º 3.340/2018 determinando a suspensão cautelar da importação, comercialização e utilização dos implantes texturizados, revogando-a em 2019, em concordância com outras agências internacionais. A empresa alegou ausência de evidência científica e apresentou plano de acompanhamento das pacientes. A manutenção das medidas tomadas pelas agências requer reavaliações da hipótese de que o implante texturizado aumenta o risco/chance de BIA-ALCL. Objetivo: Avaliar, a associação do implante mamário texturizado (IT) comparado ao liso (IL) com o risco de desenvolvimento do BIA – ALCL.

**Método:**

Foram realizadas todas as etapas de uma revisão sistemática com busca nas bases MEDLINE (PubMed), Embase e Cochrane Library com vocabulários controlados. As 563 referências identificadas foram selecionadas e avaliadas por pares de revisores independentes com divergências resolvidas por terceiro revisor. A avaliação crítica da qualidade da evidência foi realizada com a ferramenta para estudos de prevalência do Instituto Joanna Briggs. O sistema GRADE foi utilizado para avaliar a confiança na evidência.

**Resultados:**

Foram incluídas três coortes retrospectivas: 1ª) com pacientes submetidas à cirurgia de aumento mamário (266), 181 IL e 85 implantes microtexturizados, acompanhadas por média de tempo de 11 e 13 meses, respectivamente; 2ª) com pacientes submetidas à reconstrução mamária após mastectomia (389), 140 IL e 604 IT, acompanhadas por tempo médio de 234 e 221 dias; 3ª) com pacientes submetidas a reconstrução mamária após mastectomia (590), 140 IL e 450 IT, acompanhadas por tempo médio de 60 meses. Nenhuma das coortes identificou a ocorrência de BIA-ALCL, confirmando a raridade do evento. Foram encontrados vieses de seleção, aferição e evidência indireta. O tamanho das populações, possíveis fatores de confusão e tempos de seguimento insuficientes diminuíram a confiança da evidência, graduada como muito baixa a baixa. Para complementar os achados, foram avaliados dois estudos que utilizaram dados de registros nacionais e institucionais de câncer, de procedimentos de reconstrução mamária e outras bases populacionais da Suécia e Dinamarca. Respectivamente, nas populações de 3.486 e 19.885 mulheres com implantes mamários e seguimento médio de 18,4 e 7,7 anos, não foi detectado BIA-ALCL.

**Conclusão:**

A hipótese de a textura do implante mamário ser um fator de risco para o BIA-ALCL não pôde ser confirmada. As coortes comparativas foram insuficientes para garantir alguma associação. Apesar dos estudos desenvolvidos com dados de mundo real apresentarem populações maiores e longo tempo de seguimento, tais características não permitiram detectar o evento raro BIA-ALCL. O principal desafio para os estudos foram os critérios de seleção da população entre os muitos tipos de linfomas, as recorrências na mama como sítio não primário, as revisões cirúrgicas com exposições a diferentes tipos e texturas de implantes, a identificação e classificação retrospectiva dos implantes/explantes, além das mortes por doença disseminada.

